# Evaluation of Dietary Supplementation with a Blend of Organic Acids and Cinnamaldehyde on Performance and Health Status in Weaned Piglets

**DOI:** 10.3390/ani16193110

**Published:** 2026-10-03

**Authors:** Matteo Dell’Anno, Fabio Persico, Mila Meijer, Luciana Rossi

**Affiliations:** 1 Department of Veterinary Sciences, University of Messina, 98168 Messina, Italy; matteo.dellanno@unime.it; 2 Independent Researcher, 20122 Milano, Italy; 3 Perstorp Waspik BV, 5165 NH Waspik, The Netherlands; mila.meijer@perstorp.com; 4 Department of Veterinary Medicine and Animal Sciences (DIVAS), University of Milan, 26900 Lodi, Italy

**Keywords:** functional nutrition, gut health, alternatives to antimicrobials, butyric acid, propionic acid, sodium formate, phytogenic compounds

## Abstract

The post-weaning period represents a critical phase in swine production, often associated with increased disease susceptibility, impaired growth performance, and a consequent high antibiotic use. Therefore, innovative nutritional strategies based on functional feed additives are gaining increasing attention as strategies to support intestinal health and reduce the need for antimicrobials. This study evaluated the effects of dietary supplementation with a blend of organic acids and cinnamaldehyde on growth performance, health status, and antibiotic use in weaned piglets. A total of 148 piglets (Landrace × Large White; 28 ± 1 days of age) were allocated to two dietary treatments for 54 days: a control group (CTRL) receiving a standard basal diet, and a treatment group (TRT) fed the same diet supplemented with 0.1% of a blend containing propionic acid, formic acid, sodium formate, propionic acid glycerides, butyric acid glycerides, and cinnamaldehyde. Growth performance parameters were recorded, and health status was assessed through mortality, antibiotic treatments, and the occurrence of lesions. Piglets in the TRT group showed a significantly higher final body weight (*p* < 0.01) and average daily gain (*p* < 0.05) compared with CTRL, while no significant differences were observed for average daily feed intake or feed conversion ratio. Feed efficiency calculated at the pen level was not affected by dietary treatment. Importantly, the total number of antibiotic treatments was lower in the TRT group. Additionally, the incidence of auricular lesions was numerically lower in TRT compared with CTRL. In conclusion, dietary supplementation with a blend of organic acids and cinnamaldehyde improved growth performance and reduced antibiotic use in weaned piglets without negatively affecting feed efficiency. These findings highlight the potential of combining functional feed additives as a promising approach for the development of nutritional strategies aimed at supporting growth performance and health-related outcomes during the post-weaning period, while potentially contributing to a reduced need for therapeutic antibiotic interventions in swine production systems.

## Simple Summary

The period immediately after weaning is one of the most challenging stages in a pig’s life. In this moment, piglets are more prone to disease, often grow slower, and frequently antibiotic treatments are necessary to control health problems. However, the excessive use of antibiotics is a growing concern due to the development of drug-resistant bacteria, and there is increasing interest in finding alternative solutions. This study investigated whether supplementing a specific blend of natural compounds, including organic acids and cinnamaldehyde, to the diet of weaned piglets could improve their growth and health while reducing the need for antibiotics. A total of 148 piglets were divided into two groups and followed for 54 days, one receiving a standard diet (control) and the other receiving the same diet supplemented with a mixture of organic acids and cinnamaldehyde (treatment). Piglets fed the treatment diet showed increased growth and required fewer antibiotic treatments compared to those on the standard. They also showed a lower occurrence of ear lesions compared with piglets fed the control diet. No statistical differences in feed intake were observed between the experimental groups. These results suggest that innovative nutritional strategies based on the combination of organic acids and cinnamaldehyde can support animal health and reduce antibiotic use, contributing to more sustainable and responsible pig farming practices.

## 1. Introduction

The post-weaning period represents one of the most critical phases in the production cycle of swine. At weaning, piglets are exposed to multiple stressors, including abrupt dietary changes, separation from the sow, environmental rearrangement, and social regrouping, which occur in parallel with the progressive maturation of the digestive and immune systems [1]. The concomitant decline in passive immunity and limited capacity to mount an effective immune response increase susceptibility to multifactorial disorders, frequently resulting in impaired growth performance and increased morbidity [2].

Gastrointestinal disorders occurring during the post-weaning phase are among the main drivers of antibiotic use in swine production [3]. In particular, post-weaning diarrhoea (PWD) remains one of the most economically relevant conditions in pig farming, leading to reduced growth rates, increased mortality, and elevated veterinary costs [4]. Enterotoxigenic and verotoxigenic *Escherichia coli* pathotypes are recognized as major etiological agents of PWD, exploiting the physiological vulnerability of the gut to adhere to the intestinal epithelium, impair barrier integrity, and produce toxins that may lead to systemic disorders [5].

The increasing prevalence of antimicrobial-resistant bacteria has further emphasized the need for strategies capable of preserving animal health while reducing reliance on antimicrobial treatments, in line with European regulations promoting the prudent use of antimicrobials in food-producing animals (Reg. EU 2019/6) [6,7,8,9,10]

In this context, nutritional interventions targeting intestinal health represent a promising approach to improve resilience during the post-weaning period [11,12]. Diet can directly influence intestinal barrier function, microbial populations, and host immune responses [13,14,15], and functional feed additives have therefore gained increasing attention as tools to support animal health [11,15,16,17]. Among functional compounds, organic acids and phytogenic compounds represent a natural source of bioactive molecules with relevant applications in animal nutrition [18,19].

Short-chain fatty acids, particularly butyric acid, play a central role in maintaining intestinal integrity by promoting enterocyte differentiation, enhancing epithelial barrier function, and modulating inflammatory responses [20]. Dietary supplementation with butyric acid has been shown to improve gut development and reduce the incidence of post-weaning diarrhoea in piglets [20,21,22,23,24]. Propionic acid acts both as a preservative and an acidifier, contributing to feed stability and supporting intestinal eubiosis. Supplementation with propionic acid has been reported to promote intestinal health and growth performance in weaned piglets [25,26]. Diets containing blends of organic acids, including propionic and formic acids, have also been shown to alleviate immune–inflammatory responses in piglets challenged with enterotoxigenic *E. coli*, resulting in reduced diarrhoea incidence and lower circulating pro-inflammatory cytokines [26,27,28,29]. Sodium formate further contributes to these effects through its antimicrobial activity and capacity to reduce gastric pH, promoting a favourable intestinal environment and supporting beneficial microbial populations [30].

Phytogenic compounds such as cinnamaldehyde have long been used as natural preservatives due to their antimicrobial and antioxidant properties and have recently attracted renewed interest in animal nutrition. Cinnamaldehyde, alone or in combination with organic acids, has been reported to support intestinal integrity and mucosal health in piglets. In piglets experimentally challenged with enterotoxigenic *E. coli*, supplementation with blends containing cinnamaldehyde resulted in reduced intestinal lesions, increased villus height, enhanced mucosal cell proliferation, and improved intestinal antioxidant defenses [31].

Although numerous studies have investigated the effects of individual organic acids and phytogenic compounds, the available evidence remains highly variable and strongly influenced by factors such as chemical form, dosage, formulation, and delivery strategy. Moreover, most of the literature studies has been obtained under controlled experimental or challenge conditions, and uncertainty remains as to whether the beneficial effects reported for organic acids and cinnamaldehyde translate into measurable improvements in growth and health-related outcomes under commercial production conditions. Evidence regarding their potential to reduce the need for therapeutic antibiotic interventions under field conditions also remains limited.

To date, no study has specifically evaluated the in vivo effects of the specific combination of organic acids and cinnamaldehyde investigated in the present study in weaned piglets under commercial farming conditions. Therefore, the present study aimed to evaluate the effects of dietary supplementation with a specific blend of propionic acid, formic acid, sodium formate, propionic acid glycerides, butyric acid glycerides and cinnamaldehyde in weaned piglets under commercial farming conditions, with a specific focus on growth performance, health status, and antibiotic consumption as key indicators of its potential to support health and contribute to antimicrobial reduction strategies. We hypothesized that supplementation with this blend could improve growth performance and health status and reduce the need for antibiotic treatments during the post-weaning period.

## 2. Materials and Methods

### 2.1. Experimental Setting and Animal Ethics

The study was conducted as a field trial under commercial farming conditions. The experimental design complied with national and European regulations on animal welfare (Dir. EC 2008/120). The trial protocol was reviewed and approved by the Animal Welfare Organization of the University of Milan (protocol no. OPBA_113_2024, approved on 29 November 2024).

### 2.2. Animals, Housing, and Management and Experimental Diets

The trial was carried out at a commercial swine farm located in Lombardy (Italy). A total of 148 weaned piglets (Landrace × Large White), including 74 males and 74 females, were enrolled at weaning (28 ± 1 days of age) and followed for 54 days post-weaning. Piglets were allotted to the two experimental groups using a stratified randomization procedure, balancing groups for initial body weight and sex.

The housing arrangement reflected the pre-existing configuration of the commercial farm. Piglets were housed either in pens containing 5 animals or in larger pens containing 11 animals, all equipped with fully slatted floors. In the latter configuration, pairs of adjacent 11-pig pens were connected to the same feeding system and were therefore considered as a single 22-pig feeding unit for feed intake recording and the calculation of feed intake-related variables. Within each dietary treatment, the experimental arrangement comprised six 5-pig pens and two 22-pig pens, resulting in 74 piglets per treatment. All pens provided a minimum floor space of 0.50 m^2^ per piglet, 50 cm of feed trough space per piglet, and one nipple drinker for every five piglets, in accordance with Italian legislation (Legislative Decree No. 122/2011). Feed and water were provided *ad libitum* throughout the experimental period.

Environmental conditions were controlled throughout the 54-day period, with room temperature initially set at 28 °C at weaning and gradually reduced by approximately 1 °C per week in accordance with piglet growth and thermal requirements. Relative humidity was maintained at approximately 60 ± 5%.

Animals in the control group (CTRL) received a standard basal diet formulated to meet the nutritional requirements of weaned piglets [31] (NRC, 2012), whereas those in the treatment (TRT) group were fed the same basal diet supplemented with 0.1% of a blend of propionic acid, formic acid, sodium formate, propionic acid glycerides, butyric acid glycerides and cinnamaldehyde (MIX; Gastrivix™ Go, Perstorp Waspik BV). The exact composition of the MIX components is proprietary to the company Perstorp Waspik BV (Waspik, The Netherlands). Piglets were fed according to a two-phase feeding program consisting of a pre-starter and a starter diet, whose ingredient composition is presented in Table 1 and Table 2. The inclusion level used in the present study was selected in accordance with the manufacturer’s recommendations for weaned piglets.

### 2.3. Zootechnical Performance and Health Status

The experimental period lasted 54 days post-weaning. Individual body weight was recorded at the beginning of the trial (day 0) and at the end of the experimental period (day 54), and average daily gain (ADG) was calculated for each piglet based on individual weight measurements. Feed intake was recorded at the pen level throughout the entire experimental period. Based on these records, average daily feed intake (ADFI) and feed conversion ratio (FCR) were calculated for the entire experimental period. Based on the performance data, a body weight yield relative to feed intake index was calculated for each pen using the following formula:

Body weight yield relative to feed intake (%) = (total live body weight produced per pen/total feed intake per pen) × 100

Mortality and animal health were monitored daily throughout the experimental period. Piglets showing clinical signs were evaluated by the farm veterinarian, who was blinded to the dietary treatment allocation and determined the need for therapeutic intervention based exclusively on clinical assessment. When necessary, antibiotic treatment was administered under veterinary supervision. For each piglet, mortality and individual therapeutic antibiotic interventions were recorded. Treatments were categorized according to the clinical condition diagnosed by the veterinarian as enteric, respiratory, or other. Additional health-related outcomes included the frequency of piglets receiving multiple antibiotic treatments, the total number of antibiotic treatments administered during the experimental period, and the occurrence of ear lesions.

### 2.4. Data Elaboration and Statistical Analysis

An a priori sample size calculation was initially performed using G*Power 3.1. software to determine the number of individual animals to be enrolled in the study, using ADG as the reference outcome. The calculation was based on a two-tailed test for the difference between two independent means, assuming a Cohen’s d of 0.465, a significance level of α = 0.05, a statistical power (1 − β) of 0.80, and a 1:1 allocation ratio. This calculation indicated a total sample size of 148 piglets (74 animals per dietary treatment). However, this initial calculation was based on individual-animal observations and did not explicitly account for the clustering of piglets within pens. Considering the hierarchical structure of the experimental design, the dietary treatment was independently replicated across 8 experimental units per treatment (16 experimental units in total). A subsequent power assessment based on the actual number of independent treatment replicates indicated that, with 8 experimental units per treatment, α = 0.05, and a two-tailed comparison between two independent groups, the study had at least 80% statistical power to detect a large treatment effect (Cohen’s d ≈ 1.6). Prior to inferential analyses, the distribution of all continuous variables, including initial and final body weight, ADG, ADFI, and FCR, was assessed using the Shapiro–Wilk test.

Individual body weight, recorded on days 0 and 54, was analysed using a linear mixed-effects model to account for both the repeated-measures structure and the hierarchical organization of the experimental design. Treatment, time, and their interaction were included as fixed effects, while pen and individual piglet were included as random effects to account for the clustering of piglets within pens and repeated measurements within the same animal, respectively. Sex was initially included as an additional fixed effect, and the treatment × sex interaction was also tested. As neither the main effect of sex nor the treatment × sex interaction was statistically significant, these terms were therefore excluded from the final model. For piglets that died during the experimental period, subsequent unavailable body weight observations were treated as missing data in the mixed model.

ADG was calculated individually for each piglet as the difference between final and initial body weight divided by the duration of the experimental period. As ADG represented a single value per animal over the entire experimental period and therefore did not involve repeated observations within the same subject, individual ADG values were compared between dietary treatments using an independent-samples *t*-test after verification of the assumptions for parametric testing.

ADFI and FCR, which were calculated at the pen level because feed intake was recorded collectively within each pen, were also compared between treatments using independent-samples *t*-tests, with pen-level values used for the analysis. In the event of mortality, the number of animals present in the respective pen was adjusted from the day of death onward. Therefore, the deceased animal was no longer included in the calculation of feed intake per animal for the subsequent period.

Health-related variables, including mortality, antibiotic use, and the occurrence of ear lesions, were analyzed as categorical outcomes. Antibiotic administration was monitored daily at the individual-animal level throughout the experimental period. For the analysis of antibiotic use, each piglet was classified according to whether it received at least one therapeutic antibiotic treatment during the entire experimental period (0 = no antibiotic treatment; 1 = at least one antibiotic treatment), irrespective of the number or duration of treatments received. Thus, each piglet contributed only one observation to the analysis, and antibiotic use was expressed as the frequency and proportion of piglets receiving at least one therapeutic treatment. The occurrence of multiple therapeutic treatments in the same animal was evaluated separately, based on the number and proportion of piglets receiving more than one treatment during the experimental period, with each piglet again counted only once for this outcome.

Sex was additionally considered through exploratory sex-stratified analyses of the health-related outcomes. For these analyses, males and females were considered separately, and frequency distributions were compared between dietary treatments within each sex. These analyses were considered exploratory and were not interpreted as a formal assessment of a treatment × sex interaction. Frequency distributions between dietary treatments were compared using the χ^2^ test, with Fisher’s exact test applied when expected cell counts were below five. 

Statistical significance was set at *p* ≤ 0.05, while 0.05 < *p* ≤ 0.10 was considered indicative of a statistical tendency. Continuous data are reported as mean ± standard error (SE), whereas categorical data are presented as frequencies and/or percentages, as appropriate.

## 3. Results

### 3.1. Zootechnical Performance

All performance parameters showed a normal distribution, as confirmed by the Shapiro–Wilk test (*p* > 0.05), allowing the use of parametric statistical analyses.

**Figure 1:**
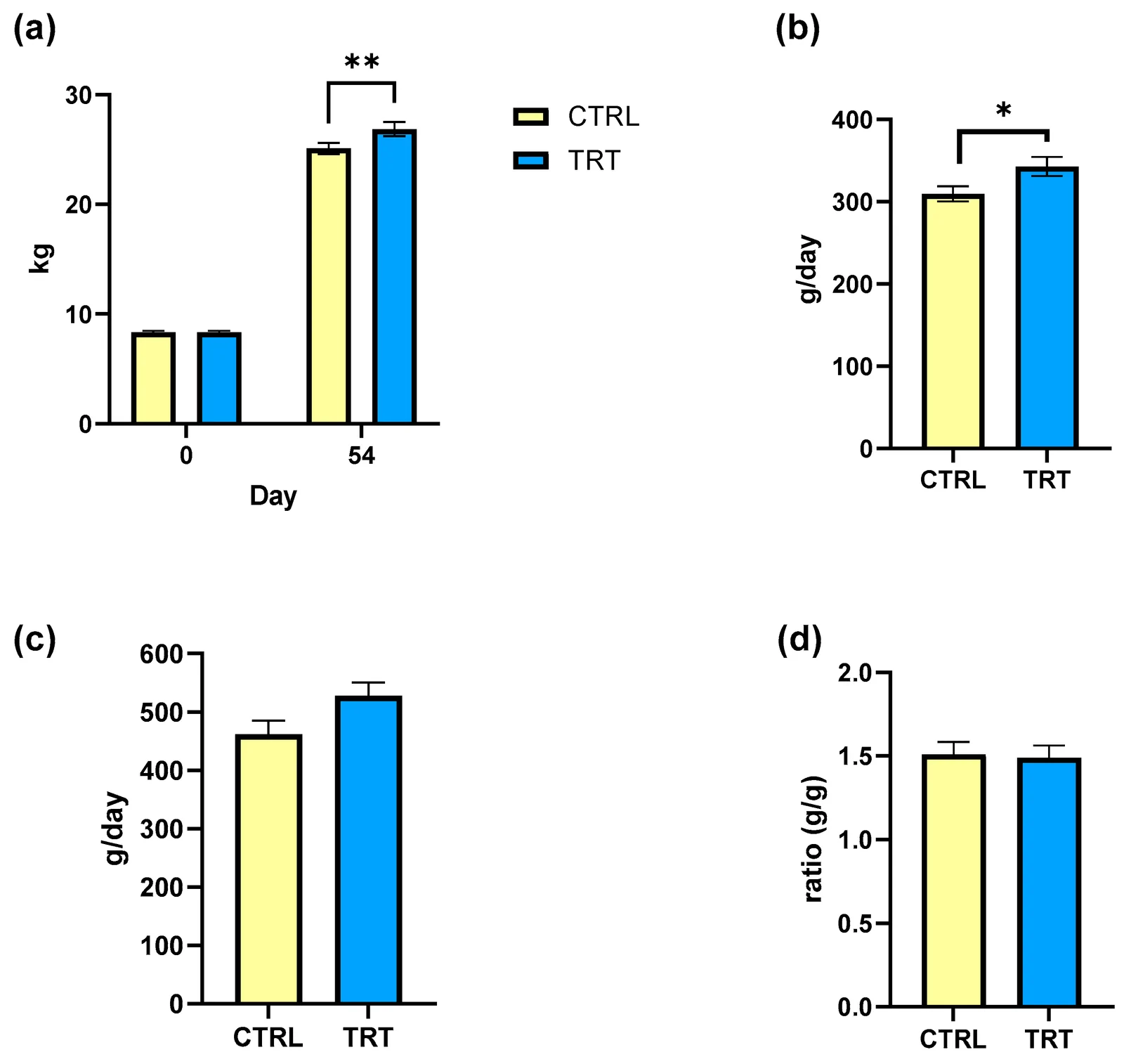
Zootechnical performance of piglets fed the control diet (CTRL) or the diet supplemented with 0.1% of the organic acids and cinnamaldehyde blend (TRT) during the 54-day experimental period. (**a**) Body weight. (**b**) Average daily gain. (**c**) Average daily feed intake. (**d**) Feed conversion ratio. Data are presented as mean ± standard error of the mean. Statistical significance between treatments is indicated as * *p* < 0.05 and ** *p* < 0.01.

Initial body weight did not differ between the two experimental groups, confirming the success of the stratified randomization procedure (Figure 1a). At the end of the 54-day experimental period, piglets receiving the TRT diet showed a significantly higher final body weight compared to the control group (26.92 ± 0.41 kg and 25.05 ± 0.41 kg, respectively; *p* = 0.0237). Consistently, ADG was significantly higher in the treated group compared to the CTRL control group (342.52 ± 11.50 g/day and 309.41 ± 9.15 g/day, respectively; *p* = 0.0260; Figure 1b).

ADFI tended to be higher in the TRT group compared with the CTRL group (528 ± 23 vs. 462 ± 23 g/day, respectively; Figure 1c; *p* = 0.0638). Similarly, no significant differences were observed between treatments for FCR (CTRL: 1.51 ± 0.07, TRT: 1.49 ± 0.07), with comparable values recorded in both groups (*p* = 0.8304; Figure 1d).

**Figure 2:**
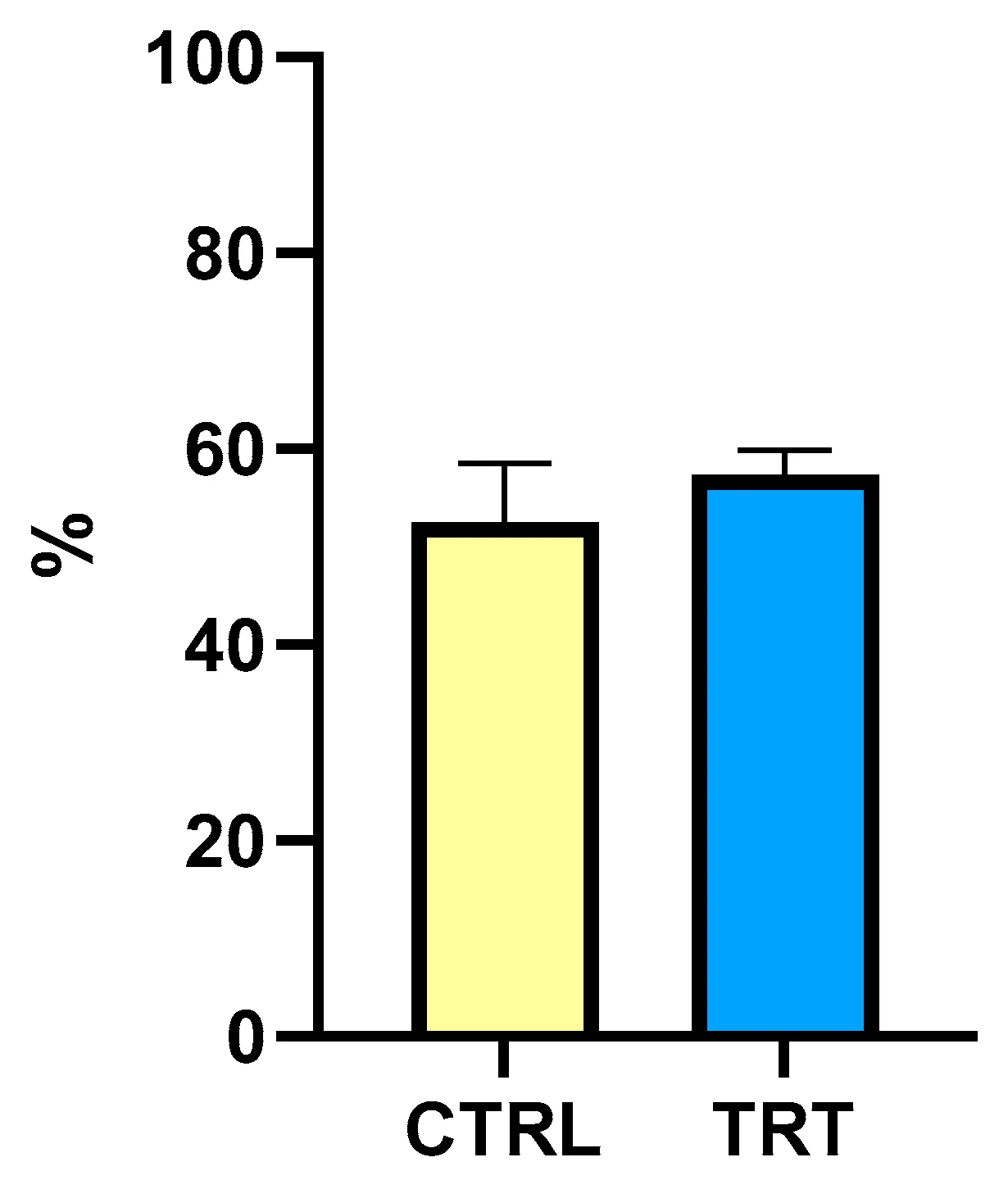
Body weight yield relative to feed intake of piglets fed the control diet (CTRL) or the diet supplemented with 0.1% of the organic acids and cinnamaldehyde blend (TRT). Body weight yield relative to feed intake was calculated at the pen level as the ratio between total live body weight produced and total feed intake. Data are presented as mean ± standard error of the mean.

The body weight yield relative to feed intake, calculated at the pen level as the ratio between total live body weight and total feed intake and expressed as a percentage, is presented in Figure 2. No significant differences were observed between CTRL and TRT groups (52.45 ± 6.11% and 57.40 ± 2.48%, respectively). Although pens receiving the dietary additive showed a numerically higher body weight yield relative to feed intake compared with the control group, this difference did not reach statistical significance (*p* = 0.4650).

### 3.2. Health Status and Antibiotic Treatments

**Figure 3:**
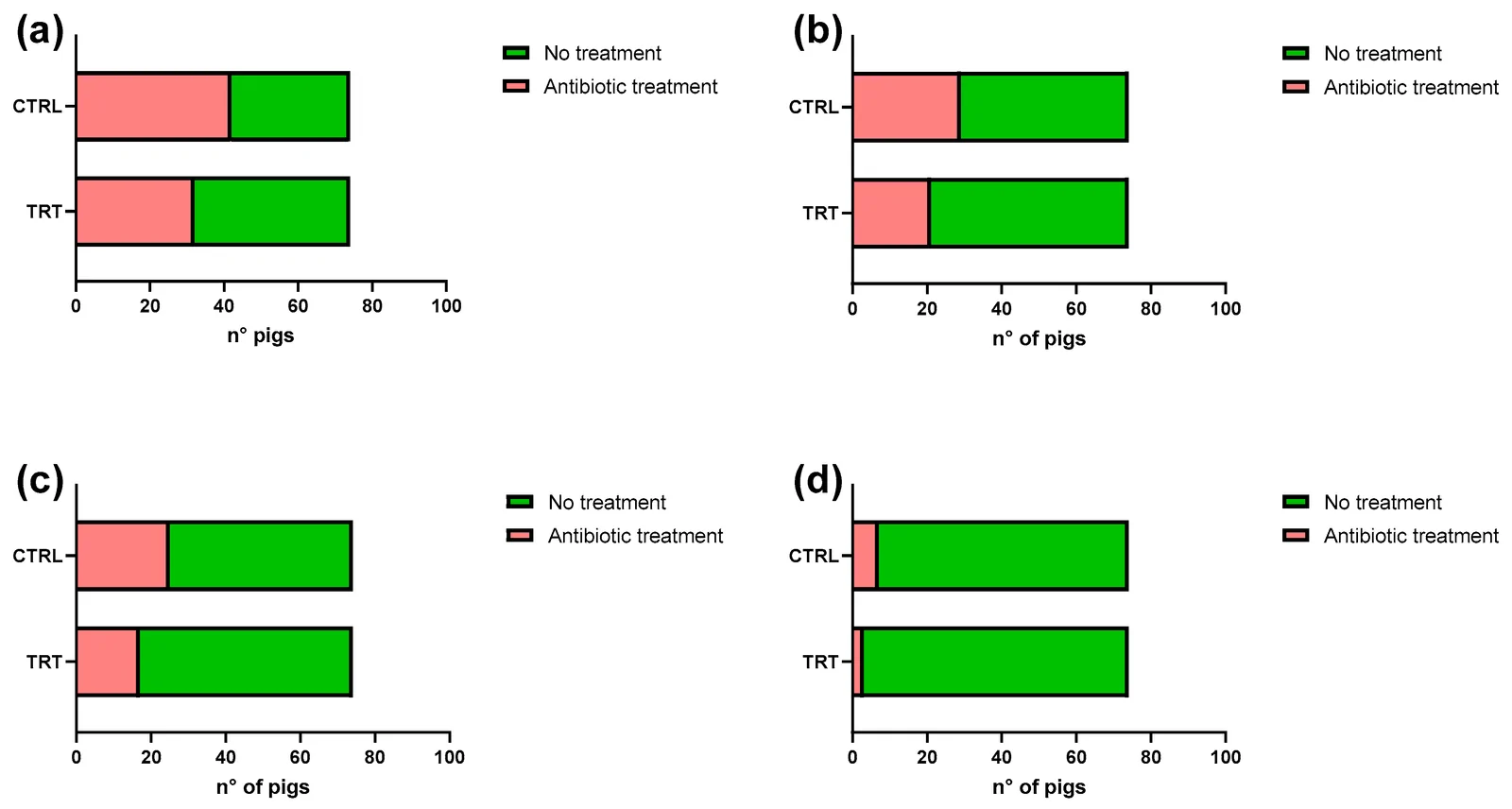
Antibiotic treatments recorded in piglets fed the control diet (CTRL) or the diet supplemented with 0.1% of the organic acids and cinnamaldehyde blend (TRT) during the experimental period. (**a**) Total number of pigs treated with antibiotic treatments. (**b**) Enteric therapies. (**c**) Respiratory therapies. (**d**) Therapies administered for other health problems. Bars represent the proportion of piglets partitioned according to antibiotic treatments and non-antibiotic treatments.

Mortality did not differ between the experimental groups over the 54-day post-weaning period (11 piglets in both the CTRL and TRT groups). Overall, 74 piglets received at least one therapeutic antibiotic treatment during the experimental period. Of these, 42 belonged to the CTRL group (42/74; 56.8%), whereas 32 belonged to the TRT group (32/74; 43.2%) (Figure 3). When antibiotic use was considered according to the clinical indication for treatment, a total of 102 category-specific treatment occurrences were recorded, with 61 in the CTRL group and 41 in the TRT group.

The total number of antibiotic treatments was 87 in the CTRL group and 50 in the TRT group, corresponding to a 42.5% reduction in the TRT group. In addition, 15 CTRL piglets and 8 TRT piglets received antibiotic treatments for more than one clinical condition, including enteric, respiratory, or other conditions, either concurrently or at different times during the experimental period.

An exploratory sex-stratified analysis showed that 24 of 39 females (61.5%) in the CTRL group and 13 of 35 females (37.1%) in the TRT group received at least one antibiotic treatment. Among males, the corresponding proportions were 18 of 35 (51.4%) and 19 of 39 (48.7%), respectively. According to the clinical indication determined by the veterinarian, 50 treatment events were associated with enteric disorders (29 CTRL and 21 TRT), 42 with respiratory disorders (25 CTRL and 17 TRT), and 10 with other clinical conditions (7 CTRL and 3 TRT).

**Figure 4:**
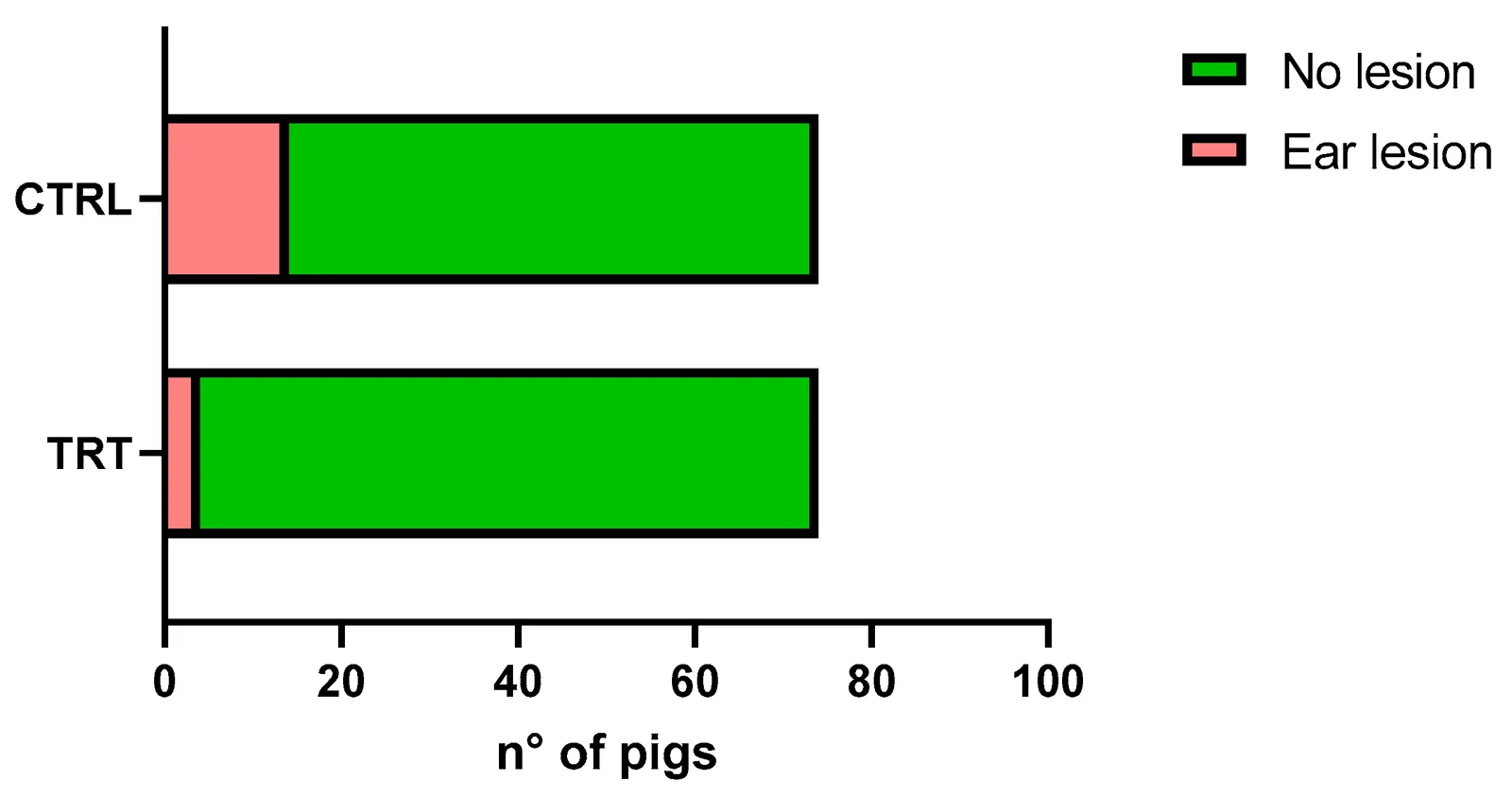
Ear lesions recorded in piglets fed the control diet (CTRL) or the diet supplemented with 0.1% of the organic acids and cinnamaldehyde blend (TRT) during the experimental period. The incidence of ear lesions was evaluated throughout the trial and expressed as the number of recorded events per treatment group.

Ear lesions were numerically less frequent in the TRT group, occurring in 4 of 74 piglets (5.4%), compared with 14 of 74 piglets (18.9%) in the CTRL group (Figure 4).

## 4. Discussion

The post-weaning phase represents a critical period in swine production, characterized by major physiological and environmental challenges that frequently impair intestinal health and growth performance [32]. In this context, the present trial evaluated the effects of dietary supplementation with an innovative blend of organic acids and cinnamaldehyde on growth performance, health status, and antibiotic use in weaned piglets under commercial farming conditions.

Piglets receiving the diet supplemented with the organic acids and cinnamaldehyde blend exhibited a significantly higher final body weight and average daily gain compared with control animals, while no differences were observed for feed conversion ratio. Although average daily feed intake did not differ significantly between treatments, it was numerically higher in the supplemented group, suggesting that the numerically higher feed consumption may have contributed to the increase in BW and ADG. Since FCR was unaffected, the observed growth response should not be interpreted as evidence of improved feed efficiency. The improvement in body weight and ADG observed in the treated group is consistent with a substantial body of literature reporting positive effects of organic acids and phytogenic compounds on post-weaning growth performance, particularly under conditions of gastrointestinal stress [33,34,35]. Several studies have demonstrated that dietary supplementation with organic acids improves growth performance by supporting gut maturation and reducing the negative impact of weaning-associated intestinal dysfunction, although the present findings do not demonstrate improved nutrient-use efficiency [36,37,38].

Organic acids such as propionic, formic, and butyric acid have been shown to promote intestinal development, enhance epithelial integrity, and modulate local immune responses, thereby improving nutrient absorption efficiency and overall growth potential in weaned piglets [20,27,39,40]. In parallel, cinnamaldehyde has been reported to exert antimicrobial and antioxidant activities that contribute to intestinal stability during the vulnerable post-weaning period, supporting growth performance particularly under commercial and stress-prone conditions [41,42].

The findings observed in the present study may be considered in the context of mechanisms previously proposed for organic acids and phytogenic compounds, although these mechanisms were not directly investigated in the current experiment. Organic acids exert their activity through both direct antimicrobial effects and indirect modulation of gastrointestinal physiology. Undissociated organic acids can penetrate bacterial cell membranes, dissociate within the cytoplasm, and disrupt intracellular pH homeostasis, leading to impaired enzyme activity and inhibition of bacterial growth. This mechanism is particularly relevant against enteric pathogens commonly involved in post-weaning disorders, including enterotoxigenic and verotoxigenic *Escherichia coli* [43,44,45].

Beyond their antimicrobial properties, organic acids may support digestive function and nutrient utilization during the early post-weaning phase, when endogenous digestive capacity is still developing. In particular, formic acid and sodium formate have been shown to reduce gastric pH, promote pepsin activation, and support a favorable intestinal environment for beneficial microbial populations [46]. These effects may collectively improve intestinal functionality and nutrient utilization, providing a plausible explanation for the higher final body weight and average daily gain observed in the treated piglets.

Short-chain fatty acids, especially butyric acid and its glyceride forms, play a central role in maintaining intestinal epithelial integrity. Butyrate serves as the primary energy source for colonocytes, promotes enterocyte differentiation, enhances tight junction assembly, and modulates inflammatory signaling pathways [24,45]. Dietary supplementation with butyric acid has been consistently associated with increased villus height, reduced intestinal permeability, and improved gut barrier function in weaned piglets, particularly under inflammatory or pathogenic challenges [47,48]. These properties are especially relevant during the post-weaning transition, when disruption of intestinal barrier function often precedes clinical disease and growth impairment.

One of the relevant findings of the present study was the numerical reduction in antibiotic use among piglets receiving the supplemented diet. The proportion of piglets receiving at least one antibiotic treatment decreased from 56.8% in the CTRL group to 43.2% in the TRT group. Moreover, the total number of antibiotic treatment events was 42.5% lower in the TRT group than in the CTRL group. Although these have to be considered as descriptive findings, the consistent numerical reduction may reflect an influence on favourable health status during the post-weaning period. However, the present study does not allow this difference to be attributed to a specific biological mechanism.

Phytogenic compounds such as cinnamaldehyde complement the activity of organic acids through additional antimicrobial, antioxidant, and anti-inflammatory mechanisms. Evidence from in vitro studies and experimental models suggests that cinnamaldehyde may disrupt bacterial cell membranes, inhibit quorum sensing, and interfere with virulence factor expression, thereby reducing pathogen colonization while exerting limited selective pressure for antimicrobial resistance [49,50,51,52]. These mechanisms provide biological plausibility for its potential contribution to the effects of the tested blend; however, they cannot be directly extrapolated to the in vivo response of weaned piglets. Similarly, the antioxidant properties reported for cinnamaldehyde may potentially contribute to mitigating oxidative processes associated with weaning stress, although oxidative status and inflammatory responses were not directly assessed in the present study.

Experimental studies in piglets challenged with enterotoxigenic *E. coli* have demonstrated that dietary supplementation with cinnamaldehyde-containing blends reduces intestinal lesions, preserves villus architecture, enhances mucosal cell proliferation, and improves antioxidant defences [31,53,54]. Collectively, these effects may reduce the severity and duration of gastrointestinal disturbances, thereby lowering the need for therapeutic antibiotic interventions.

The combination of organic acids and cinnamaldehyde may exert complementary effects through multiple mechanisms previously described in the literature, including modulation of microbial populations, support of epithelial integrity, immune-related responses, and oxidative balance. However, these potential mechanisms were not directly investigated in the present study and should therefore be considered as possible explanations rather than demonstrated effects of the tested blend. While the present study did not directly assess intestinal morphology or microbiota composition, the observed reduction in antibiotic treatments and auricular lesions indicate beneficial effects on the growth and health-related outcomes, although they should not be interpreted as direct evidence of improved intestinal health. Importantly, although the basal diets already contained functional additives commonly used under commercial farming conditions, their formulation was identical between treatments, with the addition of 0.1% MIX representing the only dietary difference between CTRL and TRT. Therefore, the observed responses can be attributed to the additional supplementation with the MIX within an established commercial feeding program.

These findings align with growing evidence supporting functional nutritional potential complementary tools for supporting animal health and prudent antimicrobial use in livestock production systems [55,56]. Rather than acting as direct substitutes for antibiotics, organic acids and phytogenic compounds may contribute to supporting animal resilience and health during critical production phases, in accordance with current European regulatory frameworks and One Health principles promoting prudent antimicrobial use (Reg. EU 2019/6).

Despite the positive outcomes observed, some limitations of the present study should be acknowledged. The experimental design and ethical authorization did not include the collection of samples for intestinal morphology, gut microbiota composition, or inflammatory and oxidative stress markers. Consequently, conclusions regarding the biological mechanisms underlying the observed responses cannot be specifically defined. Future studies integrating comprehensive biological and microbiological evaluations would be useful to further elucidate the mechanisms of action of the tested blend of organic acids and cinnamaldehyde.

## 5. Conclusions

In conclusion, under the commercial conditions examined in the present study, supplementation of the basal diet with 0.1% of the specific tested organic acid–cinnamaldehyde blend was associated with higher final BW and ADG in weaned piglets. This growth response occurred alongside a tendency toward increased ADFI in TRT, while FCR remained unaffected. Numerically fewer piglets received at least one antibiotic treatment in the supplemented group, which also showed fewer total antibiotic treatment events and ear lesions. Further studies integrating biological markers, microbiota analysis, and controlled challenge models are required to confirm these findings and elucidate the mechanisms underlying the observed responses.

## Figures and Tables

**Table 1:** Ingredient and calculated nutrient composition of the experimental pre-starter diets (%, as-fed basis).

Prestarter Diets		
Ingredients, as % of fed basis	CTRL	TRT
Barley meal	25.17	25.14
Wheat flakes	15.20	15.18
Corn flakes meal	13.00	12.99
Fermented rapeseed protein concentrate	10.00	9.99
Dehulled barley meal	7.30	7.29
Soybean protein concentrate (53% CP)	5.20	5.19
Corn meal	5.00	5.00
Biscuit meal	2.75	2.75
Coconut oil	2.00	2.00
Sweet whey	2.00	2.00
Fish meal	1.50	1.50
Dextrose monohydrate	1.50	1.50
Fermented dairy products	1.50	1.50
Acidifier mix (formic acid and sodium formate)	1.45	1.45
Soybean oil	1.00	1.00
Spray-dried plasma	1.00	1.00
Dicalcium phosphate	0.95	0.95
L-lysine	0.61	0.61
Animal fat	0.55	0.55
L-threonine	0.27	0.27
Protected benzoic acid	0.25	0.25
DL-methionine	0.22	0.22
Sodium chloride	0.12	0.12
L-Tryptophan	0.07	0.07
L-Valine	0.06	0.06
Vitamin and minerals premix ^1^	1.33	1.33
MIX ^2^	-	0.1
Calculated nutrient composition		
Crude protein (%)	16.89	16.89
Crude lipids (%)	5.74	5.74
Crude fiber (%)	3.98	3.98
Ashes (%)	4.56	4.56
DE growing pigs (kcal/kg)	3806	3806
ME growing pigs (kcal/kg)	3652	3652
NE growing pigs (kcal/kg)	2827	2827

^1^ List of additives expressed per kg of complete feed: Vitamins, pro-vitamins and chemically well-defined substances with similar effect: vitamin A (retinyl acetate, 3a672a), 15,000 IU; vitamin D_3_ (cholecalciferol, 3a671), 2000 IU; vitamin B_12_ (cyanocobalamin, 3a835), 0.03 mg; betaine hydrochloride (3a925), 750 mg; biotin (3a880), 0.20 mg; choline chloride (3a890), 400 mg; folic acid (3a316), 1.00 mg; niacinamide (3a315), 30.00 mg; calcium D-pantothenate (3a841), 15.00 mg; vitamin B_1_ (thiamine mononitrate, 3a821), 2.00 mg; vitamin B_2_ (riboflavin, 3a825i), 6.00 mg; vitamin B_6_ (pyridoxine hydrochloride, 3a831), 5.00 mg; vitamin C (sodium calcium ascorbyl phosphate, 3a312), 150.00 mg; vitamin E (all-rac-α-tocopheryl acetate, 3a700), 120.00 mg; vitamin K_3_ (menadione nicotinamide bisulphite, 3a711), 2.00 mg. Trace elements: copper (cuprous oxide, 3b412), 135.00 mg; iodine (anhydrous calcium iodate, 3b202), 1.00 mg; iron (ferrous sulphate monohydrate, 3b103), 120.00 mg; manganese (manganese(II) oxide, 3b502), 50.00 mg; selenium (sodium selenite, 3b801), 0.25 mg; selenium (selenised yeast Saccharomyces cerevisiae, 3b812), 0.10 mg; zinc (zinc oxide, 3b603), 100.00 mg. Amino acids, their salts and analogues: L-lysine monohydrochloride (3c322), 5031.00 mg; L-threonine (3c410), 2974.70 mg; L-tryptophan (3c440), 432.00 mg. Digestibility enhancers (enzymes): endo-1,3(4)-β-glucanase (EC 3.2.1.6; 4a1604i), 1500 U; endo-1,4-β-xylanase (EC 3.2.1.8; 4a1604i), 1100 U; 6-phytase (EC 3.1.3.26; 4a27), 1800 FTU. Gut flora stabilisers (probiotics): *Weizmannia faecalis* DSM 32016 (4b1900), 1.25 × 10^9^ CFU; Saccharomyces cerevisiae CNCM I-4407 (4b1702), 1.0 × 10^10^ CFU. Other zootechnical additives (performance enhancers): benzoic acid (4d210), 1232.50 mg. Preservatives: citric acid (1a330), 90.15 mg; fumaric acid (1a297), 810.00 mg; formic acid (1k236), 396.00 mg; propionic acid (1k280), 90.00 mg; calcium formate (1a238), 2657.50 mg; sodium formate (1k237i), 176.00 mg; calcium lactate (1a327), 1933.12 mg. Antioxidants: propyl gallate (E310), 0.15 mg. Binders: bentonite (1m558i), 194.00 mg; sepiolite (E562), 385.00 mg. Acidity regulators: citric acid (1a330), 128.88 mg. Flavouring compounds: *Castanea sativa* Mill., 2.00 mg; thymol (2b04006), 1.50 mg; carvacrol (2b04031), 1.50 mg. ^2^ MIX: blend of propionic acid, formic acid, sodium formate, propionic acid glycerides, butyric acid glycerides and cinnamaldehyde (Gastrivix™ Go, Perstorp Waspik BV).

**Table 2:** Ingredient and calculated nutrient composition of the experimental starter diets (%, as-fed basis).

Starter Diets		
Ingredients, as % of fed basis	CTRL	TRT
Corn flakes meal	20.00	19.98
Barley meal	20.00	19.98
Wheat flakes meal	13.27	13.26
Soybean meal	9.00	8.99
Corn meal	6.50	6.49
Wheat meal	6.50	6.49
Rapeseed fermented protein concentrate	4.00	4.00
Biscuit meal	3.25	3.25
Animal fat	2.25	2.25
Soybean hulls	2.00	2.00
Soybean protein concentrate (53% CP)	1.50	1.50
Whey fatted (50%)	1.49	1.49
Whey sweet	1.16	1.16
Fish meal	1.00	1.00
Fermented dairy products	1.00	1.00
Dicalcium phosphate	0.73	0.73
L-lysine	0.59	0.59
Dietetic fiber mix	0.50	0.50
Carob pulp	0.50	0.50
Spray-dried plasma	0.50	0.50
Protected benzoic acid	0.30	0.30
DL-methionine	0.29	0.29
L-threonine	0.29	0.29
Calcium carbonate	0.20	0.20
Sodium chloride	0.20	0.20
Acidifiers mix (formic acid and sodium formate)	0.20	0.20
Vitamin and minerals premix ^1^	2.79	2.78
MIX ^2^	-	0.1
Calculated nutrient composition		
Crude protein (%)	16.18	16.18
Crude lipids (%)	5.53	5.53
Crude fiber (%)	3.82	3.82
Ashes (%)	4.43	4.43
DE growing pigs (kcal/kg)	3420	3420
ME growing pigs (kcal/kg)	3270	3270
NE growing pigs (kcal/kg)	2490	2490

^1^ List of additives expressed per kg of complete feed: Vitamins, pro-vitamins and chemically well-defined substances with similar effect: vitamin A (retinyl acetate, 3a672a), 15,000 IU; vitamin D_3_ (cholecalciferol, 3a671), 2000 IU; vitamin B_12_ (cyanocobalamin, 3a835), 0.03 mg; betaine hydrochloride (3a925), 250.00 mg; biotin (3a880), 0.20 mg; choline chloride (3a890), 156.70 mg; folic acid (3a316), 1.00 mg; niacinamide (3a315), 30.00 mg; calcium D-pantothenate (3a841), 30.00 mg; vitamin B_1_ (thiamine mononitrate, 3a821), 2.00 mg; vitamin B_2_ (riboflavin, 3a825i), 6.00 mg; vitamin B_6_ (pyridoxine hydrochloride, 3a831), 5.00 mg; vitamin E (all-rac-α-tocopheryl acetate, 3a700), 80.00 mg; vitamin K_3_ (menadione nicotinamide bisulphite, 3a711), 2.00 mg. Trace elements: copper (cuprous oxide, 3b412), 90.00 mg; iodine (anhydrous calcium iodate, 3b202), 1.00 mg; iron (ferrous sulphate monohydrate, 3b103), 120.00 mg; manganese (manganese(II) oxide, 3b502), 50.00 mg; selenium (sodium selenite, 3b801), 0.25 mg; selenium (selenised yeast *Saccharomyces cerevisiae*, 3b812), 0.10 mg; zinc (zinc oxide, 3b603), 100.00 mg. Amino acids, their salts and analogues: L-lysine monohydrochloride (3c322), 4586.40 mg; L-threonine (3c410), 2889.00 mg; L-tryptophan (3c440), 486.72 mg. Digestibility enhancers (enzymes): endo-1,4-β-xylanase (EC 3.2.1.8; 4a1604i), 1100 U; endo-1,3(4)-β-glucanase (EC 3.2.1.6; 4a1604i), 1500 U; 6-phytase (EC 3.1.3.26; 4a27), 1800 FTU. Gut flora stabilisers (probiotics): *Weizmannia faecalis* DSM 32016 (4b1900), 1.25 × 10^9^ CFU. Other zootechnical additives (performance enhancers): benzoic acid (4d210), 1232.50 mg. Preservatives: citric acid (1a330), 527.36 mg; fumaric acid (1a297), 298.89 mg; calcium formate (1a238), 4295.68 mg; calcium lactate (1a327), 1811.42 mg. Antioxidants: butylated hydroxytoluene (BHT, E321), 0.24 mg; propyl gallate (E310), 0.12 mg. Binders: precipitated silica (E551a), 65.14 mg; bentonite (1m558i), 194.00 mg; kieselguhr (purified diatomaceous earth, E551c), 1500.00 mg; sepiolite (E562), 638.00 mg. Flavouring compounds: fumaric acid (2b08025), 25.00 mg; flavouring compounds mixture, 0.70 mg; *Castanea sativa* Mill., 1.00 mg; thymol (2b04006), 1.50 mg; carvacrol (2b04031), 1.50 mg. ^2^ MIX: blend of propionic acid, formic acid, sodium formate, propionic acid glycerides, butyric acid glycerides and cinnamaldehyde (Gastrivix™ Go, Perstorp Waspik BV).

## Data Availability

The original contributions presented in the study are included in the article, further inquiries can be directed to the corresponding author.
